# Study of the perceptions of the educational environment among undergraduate medical students of Taylor’s University

**DOI:** 10.15694/mep.2020.000046.1

**Published:** 2020-03-13

**Authors:** Win Min Thein, Roland Gamini Sirisinghe, Ihab Elsayed Mohamed Ali Abdou, Benjamin Samraj Prakash Earnest, Khine Pwint Phyu, Tint Lwin, Prabal Bhargava, Lim Yin Sear

**Affiliations:** 1 Taylor's University Malaysia

**Keywords:** Students’ perception, Educational environment, Taylor’s University Malaysia

## Abstract

This article was migrated. The article was marked as recommended.

A conducive educational environment is vital to successful learning. Perception of students about their particular educational environment may vary depending on their educational background, gender, level of study, and many other factors. Awareness and understanding of the students’ perception of educational environment are a key to improve our teaching and learning environment. The aim of the study was to determine the perception of undergraduate medical students of Taylor’s University, Malaysia on their educational environment. A cross sectional cohort study was conducted among preclinical and clinical students simultaneously at Taylor’s University, School of Medicine in July 2019. Overall total scores of perceptions (136.55 ± 19.6) and those for the subscales were very satisfactory and similar to those of other local universities as well as international universities. There was a significant difference between preclinical and clinical students in two domains; Perception of Course Organizers and Academic Self-Perception, with higher scores among clinical students in all. There was a significant difference between students with Grade ‘B’ and those with Grade ‘C’ in the mean total score and Perceptions of Atmosphere. These results, in spite of being satisfactory, urge us to seek methods of and opportunities for further enhancement of the students’ education environment.

## Introduction

The environment is defined as the setting or conditions in which a particular activity is carried on ( *Oxford Dictionaries 2018, environment*). The educational environment can be defined as the setting in which teaching and learning activities are carried on. The educational environment is said to be one of the most important determinants of an effective curriculum ( Bassaw *et al.,* 2003). The educational environment includes both the physical environment and psychological environment; the latter of which is often overlooked. Hutchinson (2003) highlighted that physical factors such as noise, classroom temperature and long sessions without a break or refreshment can make it difficult for learners and teachers to relax and pay attention. Even intrinsically motivated students can be demotivated by such external factors. Genn (2001) claimed that the learning environment is an important determinant of behaviour and that there is a recognised association between the environment and the students’ achievement. Therefore, all educators should pay attention to the role of educational environment in teaching and learning. As Roff and McAleer (2001) suggested, we believe that if we can identify and evaluate the factors influencing the educational environment and students’ and teachers’ perceptions, we can improve the environment to achieve the desired education outcomes.

### Background

Taylor’s University is one of the renowned private universities in Malaysia. It was founded in 1969 as a college and was upgraded to university in 2010. The main campus is situated in Subang Jaya, Selangor and a campus over ten thousand students are studying in many undergraduate and postgraduate programmes, one of which is the medical undergraduate programme (MBBS) in the School of Medicine. The first two years of the five-year MBBS programme constitutes the preclinical phase or ‘Phase I’. Students in Phase I are based in the main campus. The last three years are the clinical years or ‘Phase II’. Phase II students are based at a sub-campus - The Taylor’s Clinical School’ - in close proximity to the teaching Hospital, approximately 30 km from the main campus. Thus, after the first two years in Phase I, the change affecting the students is not only a change in academic content from basic sciences to clinical medicine, but also their campus life, living accommodation and social environment. The education philosophy remains consistent throughout the five years and pedagogies too, but only to a certain extent, as some differences in pedagogies between the two phases become unavoidable because of the differences in the nature of academic content between these two phases. Despite similar research have been done in some other Universities in Malaysia, our university is relatively new and preclinical campus and clinical campus are in different geographical location. This reason persuades us to look into our education environment.

## Methods

### The DREEM

The Dundee Ready Education Environment Measure (DREEM) is widely used instrument in the medical education arena ranging from America through Europe, Africa to East Asia. It is a reliable, validated inventory, a scale for measuring perceptions related to the educational environment. It claims to be generic to undergraduate health professions education ( Roff, 2005). It was developed in 1997, involved a panel of nearly 100 health professions educators from around the world and validated by over 1000 students. It contains 50 statements relating to students’ perceptions of learning, teachers, atmosphere, self-academic and social perception. Students are to respond using a 5-point Likert-type scale (0 to 4) ranging from strongly agree to strongly disagree. However, 9 of the 50 items (numbers 4, 8, 9, 17, 25, 35, 39, 48 and 50) are negative statements and should be scored 0 for strongly agree, and 4 for strongly disagree. We reminded these negative items to students in the survey form. The 50-item DREEM has a maximum score is 200 indicating the ideal educational environment ( Roff *et al*., 1997).

The inventory used in this study had the word ‘registrar’ in the original inventory changed to ‘student’.

### Methodology

#### Objective

The general objective of this study was to determine the perception of medical students at Taylor’s University, Malaysia on their educational environment using the Dundee Ready Education Environment Measure (DREEM). This included the determination of the overall score and scores for the five subscales. In addition, our specific objectives were to determine if there were any differences in perceptions based on gender, the level of study at medical school, academic performance at medical school and pre-university learning experiences. With that our research question is-

Is there any relationship between the students’ perception of educational environment and preclinical and clinical students?

### Study design

We carried out a robust literature review of all the research from different continent pertaining DREEM. The knowledge of the literature implied our conceptual framework. We assumed the relationship is a reality between gender, the level of study in medical school, academic performance at medical school and pre-university learning experiences and students’ perception of the educational environment. Based on our objective, research question, and conceptual framework, we decided to use the DREEM survey questionnaire. A cross sectional cohort study was conducted in phase I and phase II simultaneously at Taylor’s University, School of Medicine in July 2019.

### Sampling

Due to the nature to research, we used the universal sampling method. All students enrolled in the MBBS Programme were requested to participate. However, participation was voluntary. The questionnaires were distributed in paper form to all students in July 2019. The questionnaires were to be completed anonymously and without compromising students’ teaching-learning sessions. A Total of 301 questionnaires was handed out of which 278 were returned, which accounted for 92.3% of the total number of students in School of Medicine. However, during data entry, we rejected 26 forms due to incomplete answer. Therefore, total sample size is 252, male 93 and female 159. Total of 87 clinical students and 165 clinical students.

### Questionnaire

The questionnaire included instructions for answering the questions. The negatively phrased questions were pointed out so that students may pay extra attention when responding to them. In addition to the statements in the DREEM inventory, questions about the students’ gender, levels of study in medical school, level of academic achievement in medical school and pre-university education were included.

### Data analysis

The responses to the DREEM questions were coded as recommended from 0 (strongly disagree) to 4 (strongly agree). The values were inverted for the negatively phrased questions.

Students were coded into four categories based on their academic achievement in their most recent semester grading, the four categories being those students achieving A grades, B grades, C grades and below C grades.

The categorization on pre-university qualifications was based on the pre-university course attended.

The mean overall scores and the means for each subscale were determined. The mean scores of preclinical and clinical year students were compared using unpaired ‘t’ test. Similar comparison was performed based on gender. The mean scores for students at different levels of achievement in medical school and on pre-university academic qualifications were compared using one-way ANOVA with Post-Hoc tests (Tukey).

Statistical significance was set at ( *p*) value of <0.05.

Statistical analysis was performed using IBM SPSS ^®^ version 25.

## Results/Analysis

A total of 278 questionnaires was returned, of which 26 were rejected due to incomplete responses. Therefore, data from 252 valid completed questionnaires were analysed. Distribution of valid returned questionnaires according to gender and level of study is shown in Table 1 (a). The number of valid responses according to academic achievement in medical school and pre-university qualifications are shown in Table 1 (b) and Table 1 (c) respectively. Students in Semester 1 of the MBBS programme were excluded from the categorisation based on academic achievement.

**Table 1 (a).** distribution of valid returned questionnaires according to gender and level of study

	Gender	Level of Study		
	Male	Female	Preclinical	Clinical
Number of valid responses	93	159	87	165
Total	252		252	

**Table 1 (b).** distribution of valid responses according to academic achievement in medical school (Semester 1 students excluded)

Grade at most recent semester	Number
Grade A	17
Grade B	153
Grade C	49
Grade D or lower	8
Total	227

**Table 1 (c).** number of valid responses according to pre-university academic qualification

Pre-university academic qualification	Number
Foundation Studies (Malaysia)	130
Matriculation (Malaysia)	15
‘A’ Level (UK)	38
International Baccalaureate	10
Australian Matriculation	29
Monash University Foundation Year	6
Canadian Pre-University Studies	12
Others	12
Total	252

The mean total score for the DREEM inventory in our study was 136.55 ± 19.6. Table 2 shows the mean total score with the recommended interpretation of the values for the overall mean score

**Table 2.** overall mean score and recommended interpretation of overall mean score

Mean total score of current study	Recommended interpretation of mean total score( Roff, 2005).
136.55 ± 19.6	0-50 - very poor, 51-100 - plenty of problems, 101-150 - more positive than negative, and 151-200 - excellent.

The total of mean scores for the five subscales were in the satisfactory range. Table 3 shows the total mean score for each of the five subscales with the recommended interpretation of scores for each section.

**Table 3.** Mean total scores for subscales and recommended interpretation

Subscale	Mean scores in the current study	Recommended interpretation ( Roff, 2005).
Learning perception	33.54±4.5	0 - 12 very poor 13 - 24 teaching is viewed negatively 25 - 36 A more positive perception 37 - 48 learning highly effective
Teaching perception	31.13±4.9	0 - 11 bad 12 - 22 in need of revision 23 - 33 moving in the right direction 34 - 44 course organization mode
Academic self-perception	21.98±3.8	0 - 8 feeling of total failure 9 - 16 many negative aspects 17 - 24 tending to more positive 25 - 32 reliable
Atmosphere perception	32.04±5.8	0 - 12 terrible environment 13 - 24 there are aspects that need changes 25 - 36 a more positive attitude 37 - 48 a good overall perception
Social self-perception	17.83±3.8	0 - 7 wretched 8 - 14 is not a nice place 15 - 21 not so bad 22 - 28 very good

Comparison of scores, overall and for subscales between preclinical and clinical demonstrated higher scores for total and subscales in the clinical group. However, a significant difference between the two groups existed only in subscales Academic self-perception ( *p*<0.05) and Atmosphere ( *p*<0.05).

There was no difference in overall or subscale means between males and females. The scores for total, Learning perception, Teaching perception, Perception of atmosphere and Social self-perception were higher in females with only Academic self-perception higher in males, although the difference was not significant in any.

These results are shown in Table 4.

**Table 4.** comparison of overall means and means for subscales according to level of study and gender* *significantly higher than preclinical (p<0.05)*

	According to level of study		According to gender	
Domain	Preclinical	Clinical	Male	Female
Overall	131.90±19.8	138.99±19.1	135.55±21.3	137.13±18.7
Learning	32.78±4.8	33.65±4.2	33.45±4.6	33.59±4.5
Teaching	30.48±5.4	31.44±4.6*	30.51±5.7	31.50±4.5
Academic	20.87±3.7	21.98±3.6*	22.16±4.2	21.88±3.6
Atmosphere	30.56±6.0	32.30±5.2	31.86±5.9	32.15±5.8
Social	17.20±3.8	18.01±3.5	17.58±3.9	17.98±3.7

Regarding the scores for students with different grades in the most recent semester, there was a statistically significant difference between groups as determined by one-way ANOVA for mean total score (F(3, 223) =3.160, *p.* 025) and for the perception of atmosphere (F (3,223) =4.166, *p*=. 007).

A Turkey post hoc test showed that students with Grade ‘C’ had significantly lower scores than those with Grade ‘B’ in mean total score (130.14±25.6, *p*=. 025) and in the subscale Perception of atmosphere (29.82±6.9, *p*=. 007).

ANOVA did not demonstrate any significant difference between groups for mean total or subscale means to students with different pre-university qualifications.

When individual items of DREEM were considered, one item had a mean score of above 3.5, which is shown in Table 5.

**Table 5.** items with mean scores above 3.5

Item Number	Item	Mean scores
2	The course organisers are knowledgeable	3.52

Lowest scoring items: Two question items had mean scores of less than 2.0. These are shown in Table 6.

**Table 6.** items with mean scores below 2.0

Item Number	Item	Mean scores
25	The teaching overemphasises factual teaching	1.65
27	I am able to memorise all that I need	1.94

Of the 50 items, 34 had mean scores between 2.0 and 3.0. Fourteen items had scores of 3.0 and above and two had scores below 2. Table 7 gives the distribution of mean scores for individual items.

**Table 7.** distribution of mean scores for individual question items

Mean scores	Number of question items
Below 2.0	2
- < 3..0	34
≥ 3.0	14

## Discussion

The objective of this study was to determine the perception of educational environment among medical students at Taylor’s University and investigate if there is any difference in the perception between preclinical and clinical undergraduates. We also looked for any differences in perception based on demographics such as gender, the academic achievement at medical school and pre university studies. We aim to use the finding to identify aspects that may require improvement in our educational environment in all domains both in the preclinical and clinical phase. The study was carried out at an opportune time in respect of this aim, as the major curriculum review was due the following year. Since the use of DREEM has shown proven benefits in many countries and many medical universities in Malaysia, we believe the findings of this study will contribute towards identifying strengths and weaknesses in our educational environment.

### Overall scores

The overall mean DREEM score in the current study was 136.55 ± 19.6. Which falls into the ‘more positive than negative’ category. This compares favourably with results from other medical universities locally and internationally. In a similar research in Malaysia at the International Medical University (IMU) the mean score was reported to be 133.12 which is in the same category as our score, but lower ( Lai *et al*., 2009). In a study from *Universiti Sains Malaysia* (USM) the global total score was 117.9 which is considerably lower than ours ( Arzuman, Yusoff and Chit, 2010). In Universiti Sultan Zainal Abidin (UniSZA) the mean DREEM score was 128.2 for preclinical and 127.5 for clinical students both of which are lower than ours ( Rahman *et al*., 2015). A research from Management & Science University (MSU) total score was 125.3 ( Al-Naggar *et al*., 2015). While the score of this study is the highest among Malaysian medical schools, it is in the same category with all others (101-150) which is ‘more positive than negative’; the interpretation, therefore, being that all Malaysian medical schools reporting DREEM scores have opportunities to further enhance their educational environment.

Internationally, our study finding of the overall score is also within the same range with other studies in the Germany (109.25), Brazil (146.81), New Zealand (141.4 (± 15.9)) ^,^ 142.1 (± 16.2)) Pakistan (124.6 ± 21.3, 127.3 ± 19.3) Sri Lanka (108) ( Farooq *et al*., 2018; Foster Page *et al*., 2011; Jiffry *et al*., 2005; Placa *et al*., 2015; Rotthoff *et al*., 2011).

The total mean score was not significantly influence by level of study, gender or pre-university studies. There was a significant difference between students with Grade ‘B’ and students with Grade ‘C’. This seems to be due to a highly significant difference in perception of atmosphere between these two groups. We are not aware of any other study, which showed this result.

### Students’ perceptions of learning

The total mean score of this subscale (33.54±4.5) was in the “A more positive perception’ category. A result from a local university, IMU was 32.84 which is similar to our result and one from Pakistan 30.4 (± 6.2) was in the same category although somewhat lower ( Farooq *et al*., 2018; Lai *et al*., 2009). There was no difference in the mean score between pre-clinical and clinical students or between males and females. The scores were marginally higher in clinical students and in females. In spite of the fact that our preclinical and clinical students are based on two campuses with many different characteristics, learning appears to have taken place equally and satisfactorily. This indicates that there is no urgent issue that requires attention in our teaching and learning process. Students appear to be able to manage their workload effectively. The absence of a difference based on gender suggests that teaching learning processes are non-discriminatory to either gender. The mean scores were not significantly different in students with different academic achievement in medical school or different pre-university educational exposure. Similar score in all students irrespective of their grades may indicate a discrepancy between the perceived learning and actual learning among those with lower grades. Whether this is due to the learning processes provided by the School or due to an intrinsic characteristic of the lower achievers is not immediately apparent. Little information is available on this from previous studies with DREEM. Pre-university educational exposure did not have any influence on scores for this subscale perhaps most of the pre-university programs provide similar education. There is no information available on this aspect.

### Students’ perceptions of teaching

The score in this category of 31.13±4.9 in our study places it in the ‘Moving in the right direction’ category. A result from IMU is 30.50 which is within the same range while a study from result from Pakistan reported a score of 24.0 (± 5.0) in this domain ( Farooq *et al*., 2018; Lai *et al*., 2009). In our study, there was statistically significant difference ( *p* <0.05) in the mean scores between pre-clinical and clinical students with the clinical students score being higher, although both scores remain in the same category. This may be a result of higher use of more didactic modes of teaching in the preclinical phase as compared to the clinical phase. Preclinical teaching is more classroom based with lectures taking up most of the teaching time. Even more student centered modes such as problem-based learning, practical and seminars are classroom based. Clinical teaching is mainly hospital based providing closer to real life situations and experiential or situational learning which are more conducive to adult learning. However, other studies have failed to demonstrate a statistically significant difference between preclinical and clinical studies in UniSZA ( *p* =0.696) ( Rahman *et al*., 2015). The interpretation is that the teaching is acceptable at all levels of our programme but there is still room for improvement. Thus, the faculty does not need urgent retraining. There was no difference between the scores based on gender, grades or pre-university education in this domain

### Students’ academic self-perception

The mean score for academic self perception was 21.98±3.8 which is interpreted as ‘Feeling more on the positive side’. This is comparable to the finding at IMU, Malaysia (20.60) but lower than that from a study in Pakistan (22.2 ± 4.3) ( Farooq *et al*., 2018; Lai *et al*., 2009).

There was statistically significant difference ( *p*<0.05) in the mean scores between pre-clinical and clinical students in this domain. It can be naturally understood that students gain more confidence in later year of medical school. In most Asian countries students start undergraduate medical studies immediately after high school level. Naturally, it would be expected that they take more some time to adapt to the high demands of the medical programme. Students are more mature when they reach clinical tears. Moreover the nature of the subjects in the preclinical year is more of a cognitive base, whereas in clinical years is more of understanding and application, which may facilitate one’s perception of achievement. However, many studies locally and overseas failed the demonstrate a difference between preclinical and clinical students ( Rahman *et al*., 2015).

### Students’ perceptions of atmosphere

The mean score for this subscale was 32.04±5.8, which is interpreted as ‘A more positive attitude’. This is comparable to findings in other studies - result from IMU being 31.95 and from Pakistan is 31.8 ( Farooq *et al*., 2018; Lai *et al*., 2009).

There was no difference in scores in this domain between preclinical and clinical students or between males and females.

There was a statistically significant difference between the student with Grade ‘B’ and Grade ‘C’ in the most recent semester ( *p*=0.007). We postulate that the high achievers (Grade A) are deep learners and are less likely to be affected by environmental conditions and student with low grades who are surface learners ignore the effect of atmosphere. For the average student there is a significant effect of atmosphere. We found a study from India, investigating academic achievement as a factor influencing perceptions of education environment ( Miles, Swift and Leinster, 2012). They showed a difference between high achievers and low achievers. Interestingly, in our study the difference is between two groups of mid-achievers.

Pre-university studies had no influence on the scores in this domain.

### Students’ social self-perceptions

The mean score in this subscale was 17.83±3.8 in our study. This is similar to the findings in a local study from IMU (17.22) and one from Pakistan (17.6) ( Farooq *et al*., 2018; Lai *et al*., 2009). This is interpreted as ‘Not too bad’. We do not see any difference between male and female in our study, although a study in a local university, UniSZA, male students had a statistically significant ( *p*=0.001) lower social self-perception when compared to females ( Rahman *et al*., 2015). There are no differences between preclinical (17.20±3.8) and clinical (18.01±3.5) students in this domain in our study,

There is no difference among different groups based on grades and pre-university education

### Scores for individual items

For the individual questionnaires the highest mean score of 3.528 (SD 0.546) is question number 2 (the course organizers are knowledgeable). In our study this was the only item that had a score considered to be ‘excellent’ (>3.5). Several other studies have also reported this item to have the highest individual mean score. However, in these studies the responses did not place the item in the ‘excellent category. Studies with this finding include a similar study from UniSZA, Malaysia, where the highest. The mean score was also for the same item with the score of 3.39 (SD ±0. 568) and another study from Sri Lanka that had 3.26 as the mean score for the item ( Jiffry *et al*., 2005; Rahman *et al*., 2015).

In our study, two items had mean scores of less than 2.0, placing them in a ‘negative’ or ‘requiring improvement’ position. The two items were: Item 25 -.’The teaching overemphasises factual teaching’ (1.65) and Item 27. ‘I am able to memorize all I need. (1.94). A similar finding was reported in the study at UniSZA which had the lowest score of 1.67 for Item 27 ( Rahman *et al*., 2015). It is arguable whether the ability to memorize is a major necessity or even desirable in the medical curriculum. A counter argument would be that some facts require remembering and analysis and interpretation could not happen without factual knowledge. The phrase ‘.. all I need’ within the item probably supports the latter argument.

In this study, the majority of the items had scores in the ‘more positive than negative’ range. While this can be reassuring that the institution provides a reasonably conducive education environment for the students, it also indicates that there could be much more that can be done to provide an optimal environment. DREEM is not a tool that suggests how improvements can be made. It is left to the authorities and the faculty to devise possible means of improving the environment.

## Conclusion

DREEM is a well established inventory in assessing the education environment. In our study overall perception as well as all scores for each subscale was in the satisfactory range. Score were similar to but marginally higher than in most Malaysian and overseas studies. The significantly higher scores for perception of teaching and academic self-perception in clinical students are expected, although not many other studies demonstrated this. Neither gender nor pre-university studies had an effect on the scores.

Although satisfactory, the results leave room for further enhancement of the education environment of our students. While there does not seem to be any urgency to bring about major changes immediately, future curriculum reviews need to focus on the possible means of further improvement.

### Study limitations

Small sample size. The study does not represent all students from school of medicine. Being a cross sectional cohort study, we could not claim cause and effect relationship is absolutely true.

## Take Home Messages

There could be a difference in perception of educational environment among the students. We should not underestimate the impact of student psychological wellbeing pertaining their performance.

## Notes On Contributors

Win Min Thein is Phase II Program Director of School of Medicine, Faculty of Medicine and Health Sciences, Taylor’s University.

Roland Gamini Sirisinghe is Chairman of Medical Education Committee on School of Medicine, Faculty of Medicine and Health Sciences, Taylor’s University.

Ihab Elsayed Mohamed Ali Abdou is former Phase II Program Director of School of Medicine, Faculty of Medicine and Health Sciences, Taylor’s University.

Tint Lwin is a Coordinator in A&E in School of Medicine, Faculty of Medicine and Health Sciences, Taylor’s University.

Benjamin Samraj Prakash Earnest is a Coordinator in Internal Medicine in School of Medicine, Faculty of Medicine and Health Sciences, Taylor’s University.

Khine Pwint Phyu is Coordinator in Obstetrics and Gynaecology in School of Medicine, Faculty of Medicine and Health Sciences, Taylor’s University.

Lim Yin Sear is a Coordinator in Paediatric in School of Medicine, Faculty of Medicine and Health Sciences, Taylor’s University.

Prabal Bhargava is a Coordinator in Ophthalmology in School of Medicine, Faculty of Medicine and Health Sciences, Taylor’s University.
