# Supplementary material for: Study of the perceptions of the educational environment among undergraduate medical students of Taylor’s University
Source: MedEdPublish (2016). 2020 Mar 13;9:46. [Version 1] doi: 10.15694/mep.2020.000046.1 (PMC10697561; doi:10.15694/mep.2020.000046.1)
Supplement: Peer review history

# Reviewer report: Reviewer response for version 1

**DOI:** 10.21956/mep.20006.r30494

**Published:** 2020-05-24

**Authors:** Ken Masters

**Affiliations:** 1 Sultan Qaboos University

This review has been migrated. The reviewer awarded 3 stars out of 5 An interesting paper on undergraduate medical students’ perceptions of the educational environment at Taylor’s University, using the DREEM tool. Overall the paper is well-written, although the authors do need to take into account the errors and suggestions of the other reviewers.The main weakness of the paper is in the reflection of the results. The paper lays a good grounding by comparing their results to other local and international schools, but then, apart from one or two minor comments, end it there. The comparison with other schools only is a weakness, as (unless things were a complete disaster), it would be relatively easy to find studies showing a lower score. Without reference to a more detailed literature review, the authors could be accused of cherry-picking.In addition to this, however, the greatest value of the study would be in the reflection of why. It is obvious that the school has done some things right. The importance there is to reflect on why and how those things worked (so that lessons and principles could be applied elsewhere). Simultaneously, for the areas where things are not working (notably on factual learning). The authors have rather side-stepped this issue by finding a university that had a lower score than theirs (cherry-picking?) and a reader would be tempted to ask: what about all those other studies that you cited on the other points? What do they show? More worrying is the fact that the authors give the impression of simply shrugging their shoulders at this.Given that the idea of the study is to improve and strengthen, it would have been useful if the authors could have given some more pointers, rather than simply says that it is up to the authorities. Perhaps what would have strengthened the value of the research would have been qualitative work (interviews and focus groups), because, frequently the problem is not so much the activity of the school, but the students’ perception, and this may have been influenced by many things.So, I would like to see a version 2 of the paper in which some of these concerns are addressed (one cannot run the qualitative study now – perhaps that can be an opportunity for another paper).

Reviewer Expertise:

NA

No decision status is available

# Reviewer report: Reviewer response for version 1

**DOI:** 10.21956/mep.20006.r30496

**Published:** 2020-03-26

**Authors:** Deb Halder

This review has been migrated. The reviewer awarded 3 stars out of 5 Title, Abstract and ReferencingThis paper tends to make an evolutionary study between students’ perception and educational environment in order to designate how educational affects learning. But the very term ‘Study’ beginning with the title fosters redundancy of insertion as a research paper studies in general sense. Again, the clause ‘students’ perception of educational environment are a key to improve our teaching and learning environment’ uses personal pronoun ‘our’ which posits lack of impersonality of the interpretation of the results and it raises a question of ‘who’ this ‘our’ is basically targeted. The references used for this study are convincing with the aims and objectives of the study though a few references like Roff, S., McAleer, S., Harden, R.M., Qahtani, M.A., et al. (1997) are old fashioned as the environment of educational institution and their measurement change with the change of time due to rapid advancement of information and technology. Introduction and BackgroundBackground of this study presents rationale of this initiative. But this part along with introduction fails to highlight the identification of variables, positioning the research question and exhuming the hypothesis of the study. Again, the educational environment includes both the physical environment and psychological environment; the latter of which is often overlooked- this very sentence has posited two ideas which have not been justified with referencing. Again, the exposition of the paper cannot affirm what the educators should do without evaluating findings.MethodologyThe methodology section has covered almost all the criteria used as the research methods for this targeted paper. But it lacks proper order, as the title ‘Method’ precedes ‘Methodology’ which harms academic construction of a paper. The discussion under the title ‘Method’ should be research instrument. There is also confusion in sampling while it says ‘Total of 87 clinical students and 165 clinical students’. This paper does not say which scale the paper choses to interpret the result (as ratio or nominal etc.). Results and DiscussionThe results have been presented with tables and it follows categorical progression. Graphs and charts claim appropriateness for better interpretability of the results. Discussion of the result has been made on comparative study. But the intervention of various results from the institutions of various countries could have been justified previously at a literature review part of the paper so that the paper could have provided with preconception what this paper had intended to reveal. Conclusion, Implication and RecommendationThis paper does not highlight what the implication this result presents for future educational thought. Conclusion must have more analytic than descriptive and a well knitted recommendation adds strength to the wholeness of the paper. Only result is not that much important for academic research. The proper judgment for that speculation should be done through proper synchronization of all important parts which a paper must covers as crucial.

Reviewer Expertise:

NA

No decision status is available

# Reviewer report: Reviewer response for version 1

**DOI:** 10.21956/mep.20006.r30495

**Published:** 2020-03-18

**Authors:** P Ravi Shankar

**Affiliations:** 1 American International Medical University

This review has been migrated. The reviewer awarded 4 stars out of 5 Thank you for the invitation to review this manuscript. The manuscript is well-written and well organized. The authors have described their medical school and the medical program adequately. They have compared their findings with those noted at other medical schools in Malaysia and other locations. The authors could explain the pre-university academic qualifications in greater detail as they are confusing for the international reader. The authors do mention about differences in pedagogy between the preclinical and the clinical years which can be explained in greater detail. Are there any other differences in the educational set up between these two phases of the program? The authors mention small sample size as a study limitation. Can this be explained? Are there any other limitations? The quality of written English is very good. In a few places there are a few minor errors which can be corrected. This article will be of broad interest to all medical educators.

Reviewer Expertise:

NA

No decision status is available
